# Association between early blood lactate trajectories and mortality in neonates: a retrospective cohort study

**DOI:** 10.3389/fped.2026.1959433

**Published:** 2026-09-30

**Authors:** Xingxing Zhan, Yu Zhang, Hua Lu

**Affiliations:** 1 Department of Neonatology, Children’s Hospital of Chongqing Medical University, National Clinical Research Center for Children and Adolescents’ Health and Diseases, Ministry of Education Key Laboratory of Child Development and Disorders, Chongqing, China; 2 International Science and Technology Cooperation Base of Child Development and Critical Disorders, Chongqing, China; 3 Chongqing Key Laboratory of Child Rare Diseases in Infection and Immunity, Chongqing, China; 4 Department of Hospital Infection Control, Chongqing Mental Health Center, Chongqing, China; 5 Department of Pediatrics, Shapingba Hospital affiliated to Chongqing University (Shaping District People's Hospital of Chongqing), Chongqing, China

**Keywords:** association, blood lactate, mortality, neonates, trajectory analysis

## Abstract

**Background:**

Lactate is a widely used prognostic biomarker in critically ill patients, but its dynamic trajectory and prognostic value in critically ill neonates remain underexplored. This study aimed to investigate the association between dynamic lactate trajectories during the first 48 h after neonatal intensive care unit (NICU) admission and 30-day all-cause mortality risk in this population.

**Methods:**

The association between admission blood lactate levels and 30-day all-cause mortality was assessed using multivariable Cox regression and restricted cubic spline (RCS) analysis in 1,112 neonates admitted to the NICU who were included in the blood lactate trajectory classification. Blood lactate trajectories over the first 48 h were identified using a latent growth mixture model (LGMM) based on the highest blood lactate value within each 12-hour interval. A 48-hour landmark analysis was then performed, and survival follow-up was initiated at 48 h after NICU admission among neonates who were alive and eligible for trajectory classification, yielding an analysis cohort of 1,080 neonates. The association between trajectory patterns and 30-day all-cause mortality was further evaluated using multivariable Cox regression models, adjusting for potential confounders including demographics, clinical parameters, and laboratory variables. Kaplan–Meier survival curves were generated for the four trajectory groups in the landmark cohort, and differences in survival were compared using the log-rank test.

**Results:**

Admission lactate levels demonstrated a linear positive association with 30-day all-cause mortality risk (*p* for non-linearity = 0.78), with each 1 mmol/L increase associated with a 9% increase in risk after multivariable adjustment (HR 1.09, 95% CI 1.06–1.13, *p* < 0.01) in 1,112 neonates. Four distinct blood lactate trajectory patterns were identified during the first 48 h after NICU admission: a rapidly declining group (Class 1, 5.75%), a stably low group (Class 2, 88.22%), a persistently intermediate group (Class 3, 4.41%), and a persistently high group (Class 4, 1.62%). In the 48-hour landmark cohort (*n* = 1,080), compared with the stably low group, the persistently high group had the highest risk of 30-day all-cause mortality (HR 25.61, 95% CI 8.99–73.01, *p* < 0.01), followed by the persistently intermediate group (HR 6.84, 95% CI 3.39–13.78, *p* < 0.01), while the rapidly declining group also showed a significantly increased risk after full adjustment (HR 2.63, 95% CI 1.20–5.80, *p* = 0.02).

**Conclusion:**

Elevated lactate levels are associated with increased short-term mortality in neonates. Early lactate trajectories are associated with 30-day all-cause mortality and may provide additional clinically relevant information beyond a single admission lactate measurement, potentially aiding early risk stratification. Prospective multicenter studies are warranted to validate these findings.

## 1 Introduction

Neonates are defined as live-born infants from birth to 28 days of life, a critical transition period from the intrauterine to the extrauterine environment. Due to immature organ system development, neonates have limited compensatory reserve, rapid pathophysiological changes, and insidious disease progression. As specialized units for critically ill newborns, neonatal intensive care units (NICU) admit neonates with severe infections, perinatal asphyxia, congenital anomalies, and other life-threatening conditions, resulting in high complication rates and mortality risks substantially exceeding those of older children. Neonatal deaths constitute over 40% of all mortality in children under five years of age (1). In some developed countries, NICU mortality rates can reach as high as 46%, however, this figure is substantially higher in developing countries (2). Therefore, early identification of high-risk neonates and timely implementation of targeted interventions are of paramount clinical importance for improving outcomes in critically ill neonates. Currently, clinical tools for prognostic assessment primarily include neonatal mortality risk scores and conventional laboratory parameters. Regarding risk scores, the widely used Score for Neonatal Acute Physiology Perinatal Extension (SNAPPE-II) and Neonatal Critical Illness Score (NCIS) can reflect disease severity to some extent. However, these scoring systems involve cumbersome calculations and provide static cross-sectional assessments, which fail to dynamically capture disease evolution or response to therapeutic interventions. Concerning laboratory markers, traditional inflammatory indicators such as procalcitonin and C-reactive protein are widely used for disease assessment in infectious conditions. Nevertheless, their temporal changes often lag behind disease progression, and their sensitivity and specificity in predicting mortality risk remain controversial. Therefore, identifying biomarkers capable of real time monitoring and dynamic reflection of clinical deterioration is of great significance for optimizing risk stratification in critically ill neonates.

Lactate, a key byproduct of anaerobic metabolism, serves as an important indicator for assessing tissue hypoperfusion and hypoxia, with the advantages of convenient measurement and real-time dynamic monitoring (3). However, a single lactate measurement captures only the tissue perfusion status at a specific time point. With the advancement of longitudinal data analysis methods, dynamic lactate trajectories have increasingly gained attention. Compared with traditional static indicators, lactate trajectories can more precisely reflect patients’ response patterns to therapeutic interventions and the trend of disease evolution. Accumulating evidence suggests that distinct early lactate trajectories are associated with mortality risk in various critical conditions, including sepsis, shock, acute lung injury, and heart failure, and may improve risk stratification beyond single lactate measurements (4–8). Nevertheless, existing trajectory studies have predominantly focused on adult populations, and investigations regarding the association between dynamic lactate trajectories and mortality risk in critically ill neonates remain unreported. This study aims to employ a latent growth mixture model to investigate the dynamic trajectories of lactate levels during the first 96 h after NICU admission and to examine the association between distinct lactate trajectories and short-term mortality risk, thereby providing a novel reference for early risk stratification and clinical decision-making in critically ill neonates.

## 2 Design and methods

### 2.1 Data source and study population

The study utilized data from the Pediatric Intensive Care (PIC) database (version 1.1.0), a comprehensive Chinese pediatric critical care repository containing detailed clinical records of patients admitted to the Children's Hospital of Zhejiang University School of Medicine between 2010 and 2018. Patient inclusion criteria were as follows: (1) age ≤ 28 days; (2) availability of blood lactate measurements at three or more of four 12-hour time points within the first 48 h after NICU admission. Exclusion criteria included readmission to the NICU. A total of 1,112 neonates were included and were used for blood lactate trajectory classification. A 48-hour landmark analysis was then performed. Neonates who died before 48 h after NICU admission were excluded, yielding an analysis cohort of 1,080 neonates for all trajectory-based survival analyses. The patient selection process is illustrated in Figure 1. The study protocol was approved by the Institutional Review Board of the hospital (Hangzhou, China), and all procedures were conducted in accordance with the ethical standards of the Declaration of Helsinki and relevant regulations. Due to the retrospective nature of the study and the use of anonymized data, the requirement for informed consent was waived.

**Figure 1:**
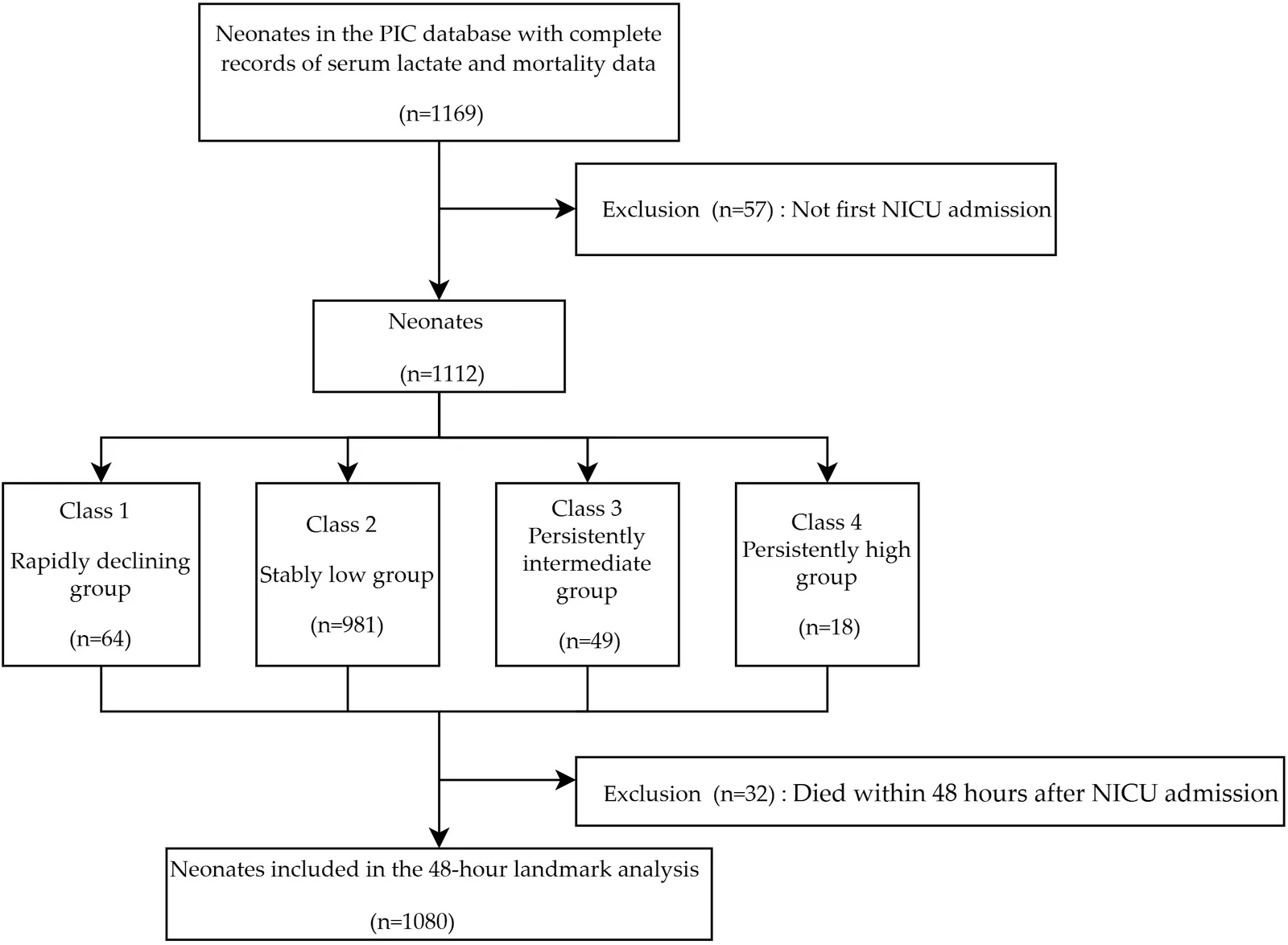
The flowchart of this study. Class 1, rapidly declining group; Class 2, stably low group; Class 3, persistently intermediate group; Class 4, persistently high group.

### 2.2 Data collection

In this study, variables were extracted from the database using structured query language (SQL). The selected covariates included demographics, vital signs, comorbidities, laboratory results and treatments. Patient baseline data were collected within the initial 24-hour period after NICU admission, and the most extreme value was selected when laboratory examinations or vital signs were measured more than once during this period. Blood lactate was measured from blood gas samples obtained during routine clinical care. For analysis of blood lactate trajectories, this study extracted the highest lactate value measured within each 12-hour interval during the first 48 h after NICU admission. Neonates with lactate measurements available at three or more of these four time points were included in the trajectory analysis. The diagnosis of comorbid conditions was based on ICD-10 classifications. The primary outcome of this study was all-cause mortality within 30 days after NICU admission. To reduce the impact of potential bias, the study excluded variables with a missing rate of more than 20%. For data with a missing rate of less than 20%, we used the ‘mice’ package of the R software and applied the random forest method of multiple imputation.

### 2.3 Statistical analysis

This study utilized R software (version 4.4.2, R Foundation for Statistical Computing, Vienna, Austria) for statistical analysis. Latent growth mixture model (LGMM) identifies subgroups with unique change patterns, can detect multiple distinct trajectories by analyzing individual change trends, thereby offering a more nuanced understanding of group heterogeneity (9). Models comprising 2 to 6 latent classes were developed, with the optimal number of classes selected based on model fit indices, including the Akaike Information Criterion (AIC), Bayesian Information Criterion (BIC), sample-size adjusted BIC (SABIC), and entropy values. A lower absolute value of BIC or AIC indicates a superior model fit. Higher values of entropy are preferable, ideally exceeding 0.8 indicating acceptable discrimination. To ensure model stability and clinical interpretability, the study required that the sample size of each latent class exceed 1% of the total study population. In addition, the average posterior probability for class membership was required to be ≥ 70% for all identified classes.

Baseline characteristics were compared across the groups. Normally distributed continuous variables are presented as mean ± standard deviation and were compared using analysis of variance (ANOVA). Non-normally distributed continuous variables are presented as median with interquartile range (25th percentile, 75th percentile) and were compared using the Kruskal–Wallis test. Categorical variables are expressed as frequencies (n) and percentages (%) and were compared using the chi-square test. The association between admission blood lactate level and 30-day all-cause mortality was assessed using a multivariable Cox proportional hazards model in all 1,112 neonates included in the trajectory classification. To explore potential nonlinear relationships between admission blood lactate level and 30-day all-cause mortality, restricted cubic splines (RCS) were employed within a Cox proportional hazards model, and the likelihood ratio test was used to compare the nonlinear model with a linear model. For trajectory-based analyses, a 48-hour landmark analysis was performed, and survival follow-up was initiated at 48 h after NICU admission among neonates who were alive and eligible for trajectory classification. A multivariable Cox proportional hazards model was used to assess the association between lactate trajectory patterns and 30-day all-cause mortality in the landmark cohort. Kaplan–Meier survival curves were generated to evaluate endpoint incidence across the trajectory groups in the landmark cohort, with the log-rank test applied to evaluate statistical differences between these groups. The results were expressed as hazard ratios (HR) with 95% confidence intervals (CI). To avoid multicollinearity, variables with a variance inflation factor (VIF) greater than 5 were excluded. All statistical tests were two-sided, and a *p*-value < 0.05 was considered statistically significant.

## 3 Results

### 3.1 Lactate trajectory classification

Based on LGMM fitting of blood lactate levels measured within each 12-hour interval during the first 48 h after NICU admission in 1,112 neonates, a four-group classification was determined to be the best fit according to statistical indices and clinical interpretability (Table 1). As the number of classes increased from two to four, AIC and BIC decreased progressively. When extending from four to six classes, the model fit did not improve further. Moreover, when extending to five or six classes, the additional classes contained very small proportions of the study population, with specific class proportions below 1%, limiting clinical interpretability and model stability. The four-class model demonstrated classification accuracy with an entropy of 0.979. The average posterior probabilities for the four classes were 0.943, 0.992, 0.960, and 0.999, respectively, confirming reliable classification. Accordingly, the 1,112 neonates were categorized into four distinct patterns (Figure 2): rapidly declining group (Class 1, 64 patients, 5.75%), stably low group (Class 2, 981 patients, 88.22%), persistently intermediate group (Class 3, 49 patients, 4.41%), and persistently high group (Class 4, 18 patients, 1.62%).

**Table 1:** Model fit indices for latent growth mixture models with 2–6 classes.

Number of classes	Log Likelihood	AIC	BIC	SABIC	Entropy	%Class 1	%Class 2	%Class 3	%Class 4	%Class 5	%Class 6
1	-9,680.103	19,374.21	19,409.30	19,387.07	1.0000000	100.000000					
2	-9,180.998	18,384.00	18,439.15	18,404.21	0.9972282	97.571942	2.428058				
3	-8,922.437	17,874.87	17,950.08	17,902.44	0.9797990	6.205036	91.366906	2.428058			
4	-8,679.606	17,397.21	17,492.48	17,432.13	0.9788981	5.755396	88.219424	4.406475	1.618705		
5	-8,679.606	17,405.21	17,520.53	17,447.48	0.9501659	0.000000	5.755396	88.219424	4.406475	1.618705	
6	-8,679.606	17,413.21	17,548.59	17,462.83	0.5681873	6.025180	0.000000	0.000000	87.859712	4.496403	1.618705

AIC, Akaike information criterion; BIC, Bayesian information criteria; SABIC, sample-adjusted information criteria; Class 1, rapidly declining group; Class 2, stably low group; Class 3, persistently intermediate group; Class 4, persistently high group.

**Figure 2:**
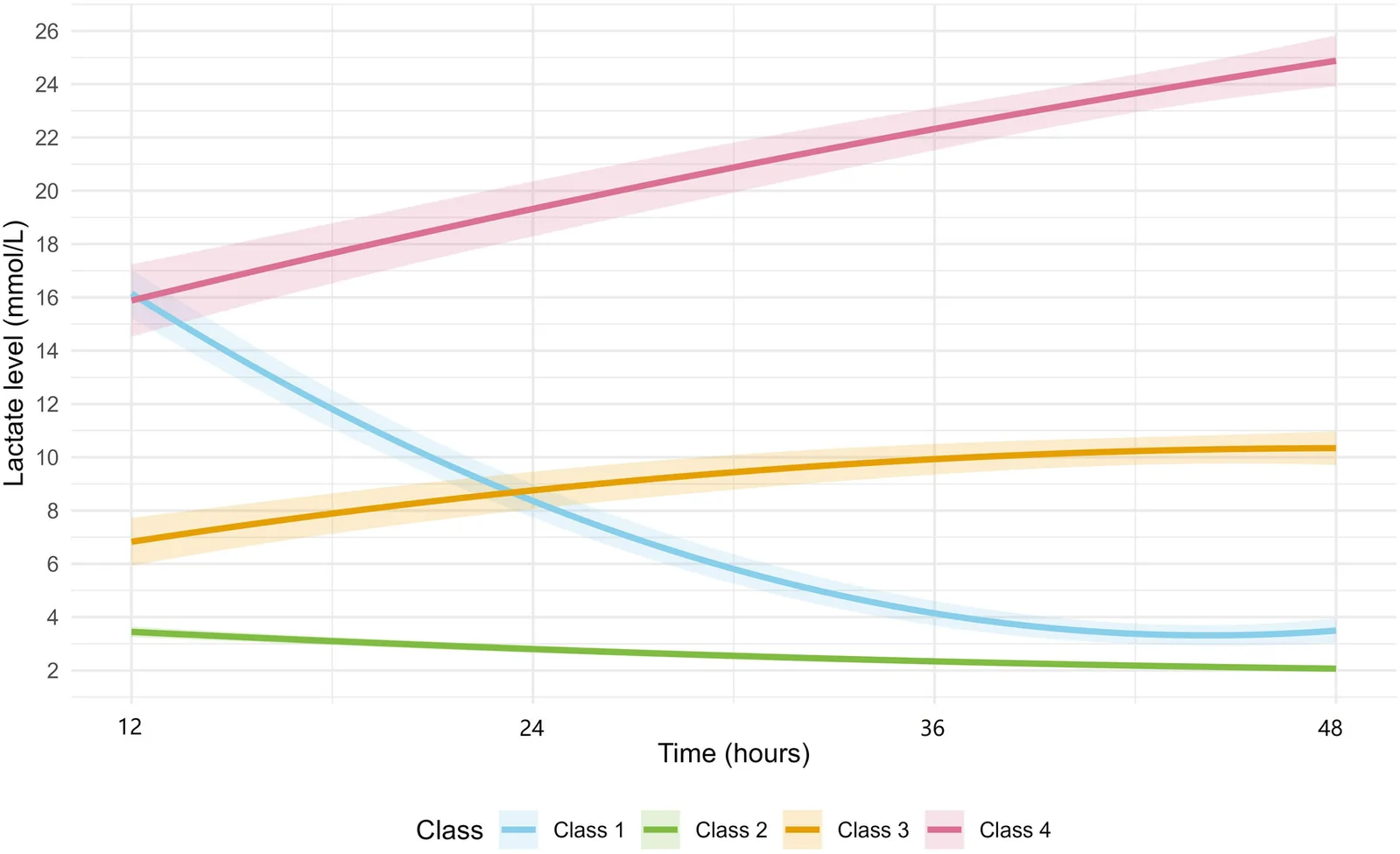
Lactate trajectories of the four latent classes during the first 48 h after NICU admission. Class 1, rapidly declining group; Class 2, stably low group; Class 3, persistently intermediate group; Class 4, persistently high group.

### 3.2 Clinical characteristics

As shown in Table 2, a total of 1,112 neonates were included and classified into four groups based on their blood lactate trajectories. No significant differences were observed among the four groups in terms of age or gender distribution. Patients in the rapidly declining group (Class 1) presented with severe clinical conditions at admission, with a relatively high incidence of sepsis and shock. Patients in the persistently intermediate group (Class 3) and the persistently high group (Class 4) exhibited more severe illness features, including higher rates of sepsis and shock, greater need for vasoactive support, and worse organ function. Notably, the 30-day mortality rate increased progressively across the four groups: 18.75% in the rapidly declining group, 7.14% in the stably low group, 44.90% in the persistently intermediate group, and 72.22% in the persistently high group. Patients in the stably low group (Class 2) had the most stable clinical indicators and the lowest mortality rate.

**Table 2:** Baseline clinical characteristics of neonates stratified by lactate trajectory groups.

Variables	Overall (*n* = 1112)	Class 1 (*n* = 64)	Class 2 (*n* = 981)	Class 3 (*n* = 49)	Class 4 (*n* = 18)	*p*-Value
Demographics						
Age, days	1.00 (0.00,6.00)	1.00 (0.00,3.25)	1.00 (0.00,6.00)	1.00 (0.00,3.00)	0.00 (0.00,1.75)	0.09
Gender (*n*, %)						0.60
Female	406 (36.51)	23 (35.94)	354 (36.09)	20 (40.82)	9 (50.00)	
Male	706 (63.49)	41 (64.06)	627 (63.91)	29 (59.18)	9 (50.00)	
Weight, kg	2.45 ± 0.95	2.64 ± 1.01	2.42 ± 0.95	2.74 ± 0.94	2.66 ± 0.84	0.03
Comorbidities						
Sepsis, (*n*, %)	51 (4.59)	6 (9.38)	39 (3.98)	6 (12.24)	0 (0.00)	<0.01
Shock, (*n*, %)	12 (1.08)	5 (7.81)	7 (0.72)	0 (0.00)	0 (0.00)	<0.01
Hepatic dysfunction, (*n*, %)	4 (0.36)	2 (3.13)	2 (0.20)	0 (0.00)	0 (0.00)	<0.01
AKI, (n, %)	221 (20.24)	14 (21.88)	193 (19.92)	10 (22.73)	4 (26.67)	0.87
NEC, (n, %)	40 (3.60)	1 (1.56)	37 (3.78)	2 (4.08)	0 (0.00)	0.67
Premature, (n, %)	286 (25.77)	11 (17.19)	265 (27.07)	9 (18.37)	1 (5.56)	0.04
Congenital heart disease, (n, %)	131 (11.80)	14 (21.88)	112 (11.44)	0 (0.00)	5 (27.78)	<0.01
MODS, (n, %)	1 (0.09)	0 (0.00)	1 (0.10)	0 (0.00)	0 (0.00)	0.99
Metabolic disorders, (n, %)	23 (2.07)	2 (3.13)	13 (1.33)	8 (16.33)	0 (0.00)	<0.01
Vital signs						
Heart rate, bpm	144.03 ± 22.10	149.03 ± 31.44	143.75 ± 21.31	140.12 ± 23.73	152.33 ± 16.57	0.06
Respiratory rate, bpm	55.45 ± 10.48	51.72 ± 11.54	55.66 ± 10.31	57.43 ± 10.46	51.89 ± 12.93	<0.01
Temperature, ℃	36.80 (36.50,37.10)	36.65 (36.25,37.03)	36.80 (36.50,37.10)	36.90 (36.30,37.10)	36.70 (36.10,36.90)	0.11
Laboratory results						
RBC, 10^12^/L	4.53 ± 0.94	4.49 ± 1.11	4.52 ± 0.91	4.73 ± 1.03	4.64 ± 1.44	0.42
WBC, 10^9^/L	15.08 (10.20,22.32)	23.59 (13.75,33.57)	14.50 (10.00,21.20)	17.17 (10.59,26.71)	23.79 (15.23,41.41)	<0.01
Platelets, 10^9^/L	237.54 ± 117.79	194.86 ± 129.59	241.99 ± 115.75	227.96 ± 137.63	173.11 ± 77.76	<0.01
Hemoglobin, g/L	164.08 ± 34.07	170.25 ± 37.28	163.30 ± 33.67	170.49 ± 34.09	166.89 ± 42.39	0.22
PCT, ng/mL	0.25 ± 0.12	0.21 ± 0.13	0.25 ± 0.12	0.22 ± 0.14	0.17 ± 0.09	<0.01
CRP, mg/L	5.00 (2.00,13.42)	4.89 (1.00,8.25)	5.00 (2.00,14.60)	6.00 (4.00,10.00)	7.00 (4.00,14.50)	0.46
ALT, U/L	12.00 (7.00,23.00)	31.00 (14.75,59.00)	11.00 (7.00,20.00)	16.00 (11.00,38.00)	43.00 (15.25,119.25)	<0.01
AST, U/L	64.00 (40.00,99.00)	110.00 (72.00,212.25)	60.00 (38.00,92.00)	86.00 (56.00,224.00)	181.50 (84.25,600.50)	<0.01
BUN, mmol/L	4.60 (3.52,6.46)	5.67 (3.96,7.34)	4.53 (3.46,6.22)	4.53 (3.69,7.27)	5.10 (3.96,8.04)	<0.01
Creatinine, μmol/L	81.00 (66.00,99.00)	100.90 (79.50,115.00)	80.00 (65.00,95.00)	101.00 (78.00,113.00)	110.50 (91.43,129.55)	<0.01
Albumin, g/L	31.42 ± 5.46	30.78 ± 6.89	31.46 ± 5.32	31.72 ± 5.66	30.63 ± 6.42	0.69
CKMB, U/L	73.00 (42.00,154.25)	270.00 (102.25,704.75)	68.00 (40.00,136.00)	98.00 (47.00,280.00)	100.00 (65.50,277.75)	<0.01
pH	7.28 (7.20,7.34)	7.15 (7.05,7.24)	7.29 (7.23,7.35)	7.20 (7.14,7.27)	7.02 (6.92,7.16)	<0.01
Lactate, mmol/L	3.70 (2.50,5.80)	15.00 (13.28,18.00)	3.40 (2.40,5.00)	7.60 (5.30,13.00)	24.00 (13.75,30.00)	<0.01
Potassium, mmol/L	4.60 (4.10,5.20)	5.15 (4.38,5.80)	4.50 (4.10,5.10)	4.80 (4.20,5.60)	5.20 (4.28,5.83)	<0.01
Sodium, mmol/L	132.00 (129.00,135.00)	130.50 (127.00,133.25)	132.00 (129.00,135.00)	131.00 (128.00,134.00)	131.50 (130.00,135.75)	0.02
PT, s	16.20 (14.10,19.40)	25.70 (19.00,38.10)	15.90 (13.80,18.80)	18.40 (15.80,29.10)	30.95 (18.55,44.40)	<0.01
APTT, s	69.05 (53.30,92.80)	80.50 (62.28,110.00)	66.60 (53.10,89.50)	67.80 (53.80,110.00)	105.10 (86.00,145.73)	<0.01
INR	1.34 (1.16,1.63)	2.14 (1.52,3.17)	1.31 (1.15,1.56)	1.50 (1.26,2.36)	2.16 (1.43,2.77)	<0.01
Interventions						
Vasoactive drug, (n, %)	168 (15.11)	17 (26.56)	143 (14.58)	7 (14.29)	1 (5.56)	0.04
Outcome						
30-day mortality, (n, %)	117 (10.52)	12 (18.75)	70 (7.14)	22 (44.90)	13 (72.22)	<0.01

AKI, acute kidney injury; NEC, necrotizing enterocolitis; MODS, multiple organ dysfunction syndrome; RBC, red blood cell; WBC, white blood cells; PCT, procalcitonin; CRP, C-reactive protein; BUN, blood urea nitrogen; CKMB, Creatine kinase-MB isoenzyme; PT, Prothrombin time; APTT, activated partial thromboplastin time; INR, international normalized ratio; ALT, alanine aminotransferase; AST, aspartate aminotransferase; Class 1, rapidly declining group; Class 2, stably low group; Class 3, persistently intermediate group; Class 4, persistently high group.

### 3.3 Association between blood lactate and risk of 30-day mortality in neonates

After adjusting for potential confounders, higher admission blood lactate levels were significantly associated with increased risk of 30-day all-cause mortality (HR 1.09, 95% CI 1.06–1.13, *p* < 0.01) (Table 3). Restricted cubic spline analysis demonstrated a linear relationship between admission blood lactate and mortality risk (overall *p* < 0.001; *p* for non-linearity = 0.78), as shown in Figure 3.

**Table 3:** Association between admission blood lactate concentration and 30-day all-cause mortality among neonates.

Variables	Crude Model HR (95% CI)	*p*-Value	Model 1 HR (95% CI)	*p*-Value	Model 2 HR (95% CI)	*p*-Value
blood lactate	1.12 (1.09, 1.14)	＜0.01	1.12 (1.09, 1.14)	＜0.01	1.09 (1.06, 1.13)	＜0.01

Model 1: unadjusted.

Model 2: Adjusted for age, gender.

Model 3: Adjusted for age, gender, weight, sepsis, NEC, congenital heart disease, premature, heart rate, respiratory rate, temprature, RBC, WBC, CRP, creatinine, BUN, albumin, potassium, sodium, CKMB, ALT, AST, APTT, INR, PT.

AKI, acute kidney injury; NEC, necrotizing enterocolitis; RBC, red blood cell; WBC, white blood cell; CRP, C-reactive protein; CKMB, creatine kinase-MB isoenzyme; ALT, alanine aminotransferase; AST, aspartate aminotransferase; APTT, activated partial thromboplastin time; INR, international normalized ratio; PT, prothrombin time; BUN, blood urea nitrogen; HR, hazard ratio; CI, confidence interval.

**Figure 3:**
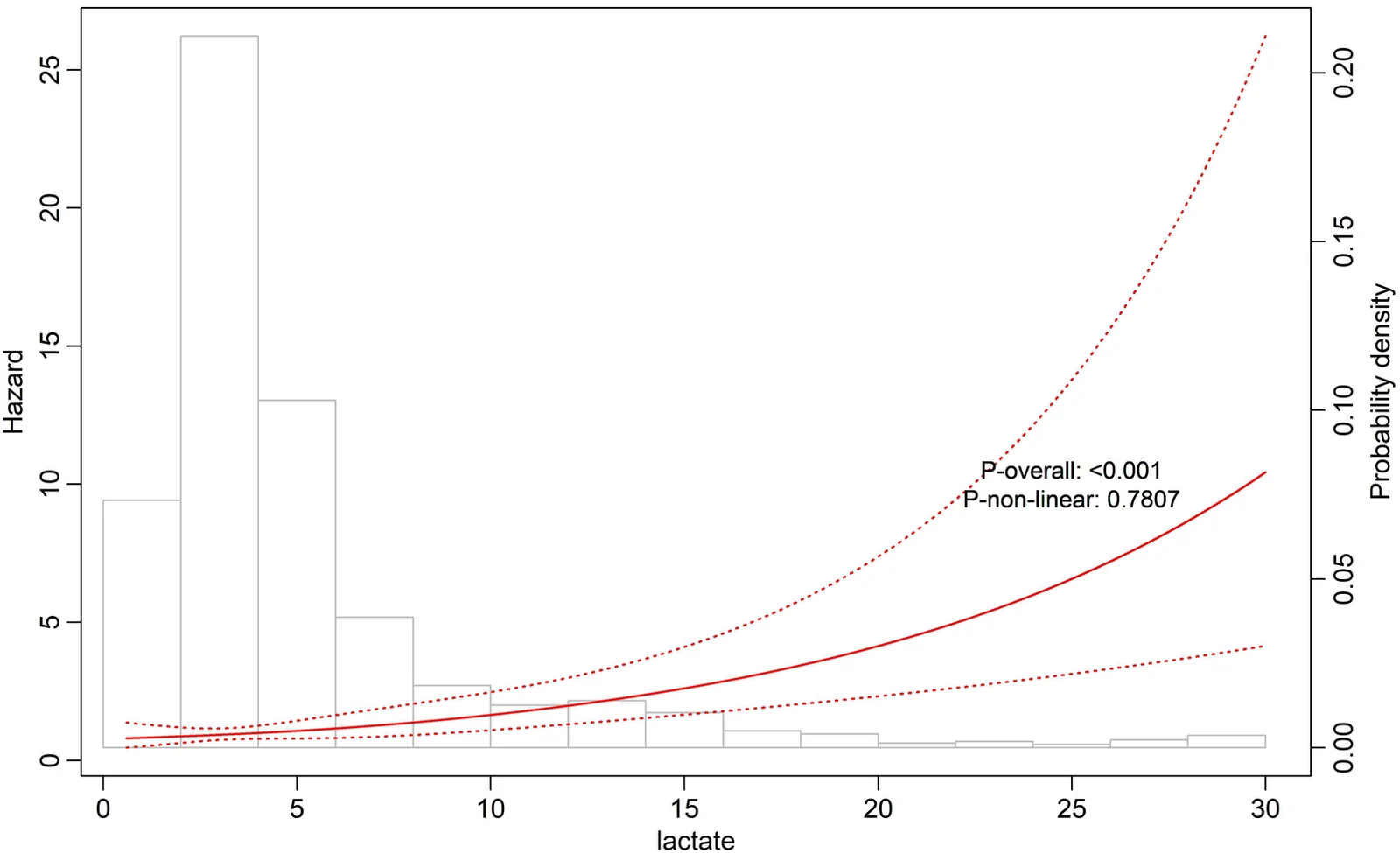
Restricted cubic spline analysis of the association between admission blood lactate level and 30-day all-cause mortality in critically ill neonates.

As shown in Table 4, after adjusting for the same set of covariates and using the stably low group (Class 2) as the reference, the persistently high group (Class 4) was independently associated with the highest risk of mortality (HR 25.61, 95% CI 8.99–73.01, *p* < 0.01), followed by the persistently intermediate group (Class 3) (HR 6.84, 95% CI 3.39–13.78, *p* < 0.01). The rapidly declining group (Class 1) also showed a significantly increased risk of mortality compared with the reference group (HR 2.63, 95% CI 1.20–5.80, *p* = 0.02). Kaplan–Meier survival analysis further revealed significant differences in survival probabilities across the four trajectory groups (log-rank *p* < 0.001). The persistently high group (Class 4) exhibited the poorest survival, with a marked decline beginning early in the follow-up period, followed by the persistently intermediate group (Class 3) and the rapidly declining group (Class 1), while the stably low group (Class 2) maintained the most favorable survival throughout the 30-day observation period (Figure 4).

**Table 4:** Association between longitudinal blood lactate trajectory patterns and 30-day all-cause mortality after the 48-hour landmark among neonates.

blood lactate trajectory	Crude Model HR (95% CI)	*p*-Value	Model 1 HR (95% CI)	*p*-Value	Model 2 HR (95% CI)	*p*-Value
Class 1	3.24 (1.75, 5.99)	＜0.01	3.25 (1.75, 6.03)	＜0.01	2.63 (1.20, 5.80)	0.02
Class 2	Reference		Reference		Reference	
Class 3	6.37 (3.44, 11.80)	＜0.01	6.44 (3.47, 11.95)	＜0.01	6.84 (3.39, 13.78)	＜0.01
Class 4	23.88 (10.32, 55.24)	＜0.01	24.75 (10.58, 57.88)	＜0.01	25.61 (8.99, 73.01)	＜0.01

Model 1: unadjusted.

Model 2: Adjusted for age, gender.

Model 3: Adjusted for age, gender, weight, sepsis, NEC, congenital heart disease, premature, heart rate, respiratory rate, temprature, RBC, WBC, CRP, creatinine, BUN, albumin, potassium, sodium, CKMB, ALT, AST, APTT, INR, PT.

AKI, acute kidney injury; NEC, necrotizing enterocolitis; RBC, red blood cell; WBC, white blood cell; CRP, C-reactive protein; CKMB, creatine kinase-MB isoenzyme; ALT, alanine aminotransferase; AST, aspartate aminotransferase; APTT, activated partial thromboplastin time; INR, international normalized ratio; PT, prothrombin time; BUN, blood urea nitrogen; HR, hazard ratio; CI, confidence interval; Class 1, rapidly declining group; Class 2, stably low group; Class 3, persistently intermediate group; Class 4, persistently high group.

**Figure 4:**
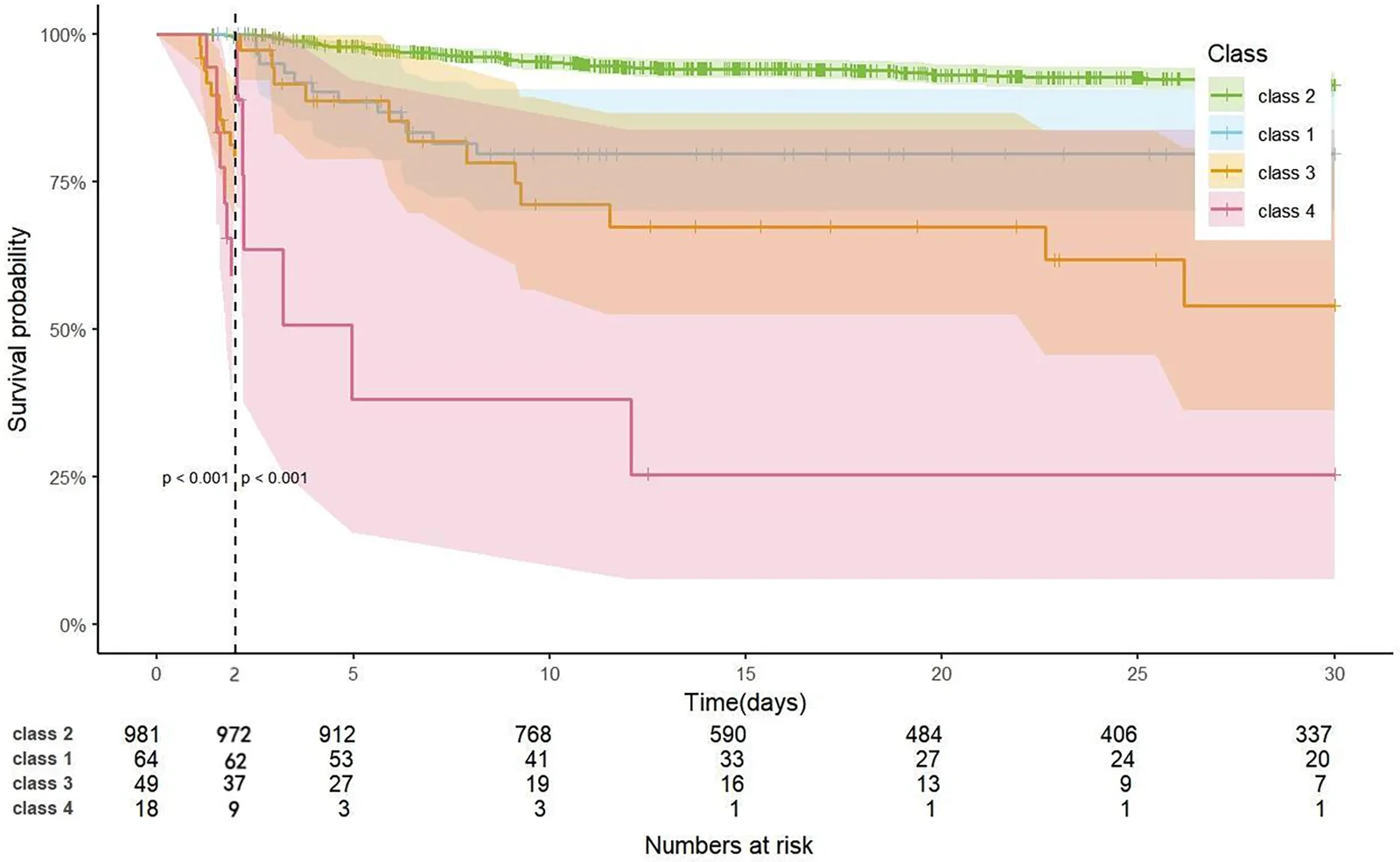
Kaplan–meier survival curves for 30-day all-cause mortality after the 48-hour landmark according to blood lactate trajectory groups. Class 1, rapidly declining group; Class 2, stably low group; Class 3, persistently intermediate group; Class 4, persistently high group.

## 4 Discussion

This study is the first to employ a latent growth mixture model to investigate the association between dynamic blood lactate trajectories during the first 48 h after NICU admission and 30-day all-cause mortality risk in neonates. The results showed a linear positive association between admission blood lactate levels and mortality risk. Based on blood lactate trajectories, patients were classified into four groups: a rapidly declining group (Class 1, 5.75%), a stably low group (Class 2, 88.22%), a persistently intermediate group (Class 3, 4.41%), and a persistently high group (Class 4, 1.62%). Using the stably low group as the reference, the persistently high group had the highest risk of mortality (HR 25.61, *p* < 0.01), followed by the persistently intermediate group (HR 6.84, *p* < 0.01). The rapidly declining group also show a statistically significant increase in risk after full adjustment (HR 2.63, *p* = 0.02). These findings suggest that dynamic blood lactate trajectories may facilitate early identification of high-risk neonates and may provide additional clinically relevant information beyond a single admission blood lactate measurement.

Previous studies have demonstrated that elevated admission lactate levels are closely associated with adverse outcomes in various critical neonatal conditions, including preterm birth, birth asphyxia, necrotizing enterocolitis, neonatal sepsis, and congenital heart disease (10–16). For instance, a cross-sectional study of term neonates with birth asphyxia reported that lactate levels ≥10 mmol/L were strongly associated with a higher risk of death (17). Additionally, Oh et al. employed machine learning to evaluate the prognostic value of lactate in very low birth weight preterm infants and found that lactate levels within the first 12 h of life ranked among the top five most important predictors of in-hospital mortality (18). However, a single lactate measurement captures tissue perfusion status at only one time point, failing to reflect dynamic changes in disease progression. In recent years, dynamic lactate trajectories have been increasingly recognized as associated with prognosis in critically ill adults and may provide additional information on disease evolution and treatment response (6, 7). In pediatric patients with severe sepsis, prospective cohort studies have shown that lactate dynamics and lactate clearance are associated with mortality and organ dysfunction, including 6-hour lactate clearance in severe sepsis or septic shock and 24-hour lactate clearance in pediatric septic shock (19, 20). Nevertheless, studies investigating the association between dynamic lactate trajectories and mortality risk in critically ill neonates remain absent. The present study is the first to apply a latent growth mixture model to analyze lactate trajectories in neonates, thereby addressing this research gap and providing a novel basis for early risk stratification in critically ill neonates.

Lactate is a key byproduct of anaerobic metabolism, and elevated levels reflect an imbalance between lactate production and clearance. Tissue hypoxia leading to impaired oxidative phosphorylation and compensatory enhancement of anaerobic glycolysis is the most common cause of hyperlactatemia (21). In addition, stress-induced release of catecholamines can stimulate excessive glycolysis in skeletal muscle, further contributing to lactate production (22). The liver and kidneys are the primary organs responsible for lactate metabolism. Thus, hepatic or renal dysfunction may impair lactate clearance. Furthermore, mitochondrial dysfunction can also result in elevated lactate levels (21–23). Consequently, hyperlactatemia is not attributable to a single factor but arises from the interplay of multiple pathophysiological mechanisms. In critical illness, conditions such as shock, severe infection, and asphyxia can lead to systemic tissue hypoperfusion, with compensatory anaerobic glycolysis driving excessive lactate accumulation. Beyond reflecting the severity of tissue hypoxia, hyperlactatemia may further promote the development of multiple organ failure through mechanisms including myocardial depression, induction of inflammatory responses, and exacerbation of mitochondrial dysfunction (23–25). Neonates exhibit unique physiological characteristics of lactate metabolism that enhance the prognostic value of lactate in this population. First, the neonatal myocardium is immature, characterized by weak contractility and limited cardiac output reserve, resulting in a substantially lower capacity to compensate for hypoxia compared with older children and adults. Even mild tissue hypoperfusion may rapidly progress to circulatory failure (26). Second, hepatic and renal function are not fully developed in neonates, leading to impaired gluconeogenesis and reduced lactate clearance capacity, which predisposes to lactate accumulation and delayed elimination. Third, the immature lungs of neonates restrict oxygen exchange, leaving limited compensatory capacity via anaerobic metabolism during hypoxic stress. Moreover, various critical illnesses in the neonatal period, such as perinatal asphyxia, sepsis, and congenital heart disease, can directly or indirectly induce tissue hypoxia and hyperlactatemia, and the dynamic changes in lactate levels precisely reflect the pathophysiological evolution of these conditions. Therefore, in critically ill neonates, lactate level is not only a sensitive marker of tissue perfusion status but also a comprehensive indicator of disease severity and physiological reserve. The dynamic trajectory of lactate holds significant clinical value for early identification of high-risk neonates and prediction of mortality.

Using a latent growth mixture model, four distinct blood lactate trajectory patterns were identified within the first 48 h after NICU admission. These groups differed significantly in clinical characteristics and 30-day all-cause mortality. The persistently high group (Class 4) showed sustained extreme hyperlactatemia, rising from approximately 16 to 25 mmol/L, with a 30-day all-cause mortality of 72.22%. In the 48-hour landmark analysis, this group had the highest mortality risk. This pattern may indicate sustained tissue hypoperfusion that is difficult to reverse, and mechanisms such as microcirculatory dysfunction, refractory shock, or mitochondrial impairment are plausible. Despite active treatment, the pathophysiological state may not improve, and continued lactate accumulation may precede death. For neonates with persistently elevated or rising blood lactate within 48 h after admission, high vigilance, escalation of treatment intensity, and early evaluation for extracorporeal life support are warranted while underlying causes are addressed. The number of neonates in this group was relatively small at the 48-hour landmark, and the corresponding confidence interval was wide. Further validation in larger cohorts is therefore warranted. The persistently intermediate group (Class 3) maintained intermediate lactate levels, rising from approximately 7 to 10 mmol/L within 48 h, an increase of approximately 50%, with a 30-day all-cause mortality of 44.90%. In the 48-hour landmark analysis, this group had a significantly higher mortality risk than the stably low group. This pattern suggests unresolved tissue hypoperfusion and ongoing disease progression, identifying these neonates as a high-risk group requiring close monitoring and active intervention. Notably, single lactate values may not be markedly elevated in this group, but a persistently non-decreasing trajectory is easily overlooked and warrants early identification. The rapidly declining group (Class 1) showed a distinct clinical course. These neonates were more severely ill at admission, with extremely high blood lactate (approximately 16 mmol/L) and a relatively high incidence of sepsis and shock, but lactate declined by more than 50% within 48 h to near-normal levels, and 30-day all-cause mortality was 18.75%. In the 48-hour landmark analysis, this group still had a significantly higher mortality risk than the stably low group. These findings suggest that extremely high admission lactate does not necessarily indicate an irreversible poor prognosis, but a rapid decline does not equate to complete recovery. Premature pessimistic prognostication based on initial hyperlactatemia should be avoided, and residual risk after 48 h should continue to be monitored. The stably low group (Class 2) had the most favorable prognosis, with low and stable blood lactate levels and a 30-day all-cause mortality of only 7.14%. This trajectory reflects adequate tissue perfusion and effective compensatory capacity, suggesting either milder disease severity or a favorable response to standard treatment with stable hemodynamic status. These findings provide new insights into the clinical management of critically ill neonates. First, dynamic blood lactate trajectories may provide additional clinically relevant information beyond a single blood lactate measurement, enabling more precise identification of high-risk neonates and avoiding premature pessimistic prognostication in those with initially high but reversible hyperlactatemia. Second, clinical decision-making should focus on treatment responsiveness, with close monitoring of blood lactate trends. For neonates with persistently elevated or rising lactate despite treatment, heightened vigilance and timely adjustment of therapeutic strategies are warranted. Third, the marked differences in mortality risk across trajectory groups suggest that trajectory-based early risk stratification may have potential clinical utility. Fourth, the persistent residual risk observed in the rapidly declining group after the 48-hour landmark supports continued monitoring beyond the initial 48 h. However, this was an observational study, and these findings reflect associations rather than causation. The proposed trajectory patterns and approximate thresholds have not been externally validated, and standardized clinical cutoff values for defining trajectory groups have not yet been established. Therefore, generalization of these findings to other neonatal populations remains limited, and further validation in prospective multicenter studies is warranted before they can be applied in routine clinical practice.

This study is the first to apply the latent growth mixture model to analyze dynamic blood lactate trajectories in critically ill neonates, moving beyond previous studies that focused solely on single lactate measurements, thereby providing a novel dynamic perspective for prognostic assessment in this population. However, several limitations should be acknowledged. First, survivor and selection bias may still persist. In this study, blood lactate trajectory classification required neonates to have blood lactate measurements at three or more of four 12-hour time points within the first 48 h after NICU admission. Therefore, neonates who died extremely early, were too critically ill to complete at least three blood lactate measurements, or were transferred early may still have been excluded. This may have led to underestimation of the true mortality risk and may have attenuated the association between high-lactate trajectories and mortality risk. In addition, trajectory groups were derived using a data-driven latent growth mixture model, and the persistently high and persistently intermediate groups were small. As a single-center retrospective cohort, the findings require validation in larger, multicenter prospective cohorts before generalization to other neonatal populations. Second, the frequency of blood gas lactate measurements differed across trajectory groups, with sicker neonates undergoing more frequent testing. Because trajectories were constructed using the highest blood lactate value within each 12-hour interval, this differential sampling intensity may have introduced information bias, as more frequent measurements could increase the likelihood of detecting higher lactate values. It should be considered when interpreting the trajectory patterns. Third, blood lactate values were extracted from blood gas analysis records, but the PIC database labeled specimens only as “blood” and did not specify the sample source or the analytical platform. These unrecorded technical factors may have influenced measured lactate values and introduced measurement bias. Fourth, due to limitations inherent to the PIC database, which covers patients of all ages rather than a dedicated neonatal population, key perinatal variables such as gestational age, birth weight, Apgar scores, and maternal or antenatal risk factors could not be obtained. Important confounders such as continuous renal replacement therapy or hemodialysis were also unavailable. Available proxy variables including admission weight, age at admission, and a diagnosis of prematurity were used, but residual confounding may remain. Despite these limitations, this study is the first to demonstrate the association between dynamic blood lactate trajectories and 30-day all-cause mortality in critically ill neonates, offering a new evidence-based reference for early identification of high-risk neonates and implementation of individualized interventions.

## 5 Conclusions

Elevated blood lactate levels are associated with an increased risk of short-term mortality in critically ill neonates. Dynamic lactate trajectories offer greater prognostic value than single measurements, with treatment responsiveness playing a key role in risk stratification. Future prospective multicenter studies are warranted to further validate these findings.

## Data Availability

The original contributions presented in the study are publicly available. This data can be found here: https://www.physionet.org/content/picdb/1.1.0/.
